# 66. Macrophage activation syndrome: a fatal complication in a patient with SLE

**DOI:** 10.1093/rap/rky034.029

**Published:** 2018-09-20

**Authors:** Vishal Kakkar, Nikolaos Dadiras, Bela Szebenyi, Tim Gillott, Amir Alvi

**Affiliations:** Rheumatology, Diana Princess of Wales Hospital, Grimsby, UNITED KINGDOM

**Introduction:** Macrophage activation syndrome (MAS) is a potentially fatal condition belonging to the haemophagocytic lymphohistiocytosis (HLH) group of diseases. HLH is divided into familial HLH (fHLH) and secondary or acquired HLH (sHLH). MAS most frequently occurs in patients with juvenile idiopathic arthritis (JIA), but it can also arise as a complication of several other systemic autoimmune disorders, including systemic lupus erythematosus (SLE), rheumatoid arthritis (RA) and adult onset Still’s disease (AOSD), as well as in malignancy and infection. When sHLH occurs in the presence of a pre-existing autoimmune condition, it is designated as MAS. MAS is a hyperinflammatory condition, in which there is an uncontrolled activation and proliferation of T-lymphocytes and macrophages leading to a cytokine storm, eventually causing multi-organ failure. Retrospective studies have shown that MAS is under-recognised, which highlights the need for greater awareness and understanding of the condition.

**Case description:** We present a case of a 39-year-old female patient with a history of SLE, who presented to A&E with right upper quadrant pain, pyrexia and lethargy. Two weeks prior to admission she had had a dental abscess drained and had completed a course of antibiotics for this. Now she was suspected to have sepsis secondary to cholangiitis as her CRP was 120 mg/L and her white cell count (WCC) 1.7x10^9^/L with neutrophils of 1.39x10^9^/L. Her liver function tests were deranged with an ALT of 167 U/L, ALP 417 U/L, bilirubin 101 umol/L and normal renal function. She was primarily admitted by our surgical team. A CT scan of the abdomen and pelvis was performed showing submucosal oedema of the gallbladder wall, hepatitis and mild hepato-splenomegaly, but no sign of cholecystitis. At this point she was transferred to the high dependency unit and her bloods repeated, which showed the development of pancytopenia with a haemoglobin level of 86 g/L, WCC of 1.2x10^9^/L with neutrophils of 0.91x10^9^/L and platelets of 39x10^9^/L. Her liver function had also continued to worsen. With her SLE, she was normally on azathioprine and hydroxychloroquine and these were stopped. She was commenced on intravenous antibiotics for neutropenic sepsis and oxygen, as a blood gas results revealed a type-1 respiratory failure. She was reviewed by the intensivists at this point who agreed for the transfer to ITU. Once in ITU, it was decided to inform the rheumatology department and our consultant was contacted. We asked for an urgent ferritin level and LDH to be performed, and this revealed a ferritin level of 23333 ug/L and an LDH of 576 U/L. She was also found to have a hypo-complementaemia, and her CRP was high at 62 mg/L with a near-normal ESR of 14 mm/h. She was diagnosed with suspected MAS after an H-score was performed showing an 85% probability of the diagnosis being MAS and immediately was given 750 mgs of IV methylprednisolone. Blood gas results now showed a type-2 respiratory failure and she was commenced on non-invasive ventilation. A CT thorax was requested which revealed bilateral lower lobe consolidations with small effusions. She was changed to broad-spectrum antibiotics and she was set for three doses of IV methylprednisolone in total, with a switch to IV hydrocortisone thereafter. Her condition continued to deteriorate in ITU, and she was now intubated and requiring vasopressor support. Her blood tests showed a persistent pancytopenia and a ferritin elevated to 53616 ug/L. A discussion with the haematologists to perform a bone marrow biopsy then occurred, who then proceeded with this procedure. She was also given blood and platelet transfusions. Unfortunately the patient’s condition had deteriorated, resulting in progressive multi-organ failure and leaving no room for further specific therapeutic approaches, such as anakinra or etoposide. Finally, she sadly passed away. The bone marrow aspirate later confirmed prominent haemophagocytosis in keeping with the diagnosis of MAS.

**Discussion:** Diagnosing MAS can be difficult, and initial blood results are vital. A persistently high CRP in combination with a normal or near normal ESR with thrombocytopaenia in a febrile patient are the early signs of an active rheumatological condition. Other features such as a raised D-dimer, hyperferritinaemia, pancytopaenia, deranged liver function tests, hypertriglyceridaemia and coagulopathy are characteristic of MAS. A bone marrow biopsy can confirm the diagnosis in the presence of haemophagocytosis, although this is considered a late feature of sHLH. In our case the patient had the majority of the cardinal features of MAS, and it is possible that the dental infection was the trigger. MAS is most prevalent in patients with AOSD, but as SLE is more common, there are more reported cases of SLE-associated MAS. SLE patients admitted to hospital with fever were the subject of a large retrospective trial in 2016 applying the sJIA PRINTO Classification Criteria for MAS. The study found that of these patients one-third were classified as having MAS, of whom 35% died. Overall mortality itself for autoimmune disease-associated MAS ranges from 5% to 39%. The H-score was developed by Fardet et al in 2014, which integrates nine key clinical and laboratory features of HLH to produce a probability score of sHLH. Our patient scored 85% on this. At this point she was commenced on IV methylprednisolone. On reviewing the current literature, they would recommend giving a higher dose of IV methylprednisolone than we did at 1g daily and also giving IV immunoglobulin, anakinra or etoposide.

**Key Learning Points:** Macrophage activation syndrome mortality has been found to be 39% in some studies, therefore a greater awareness of this condition is imperative. Further key learning points from this case include the diagnostic criteria of MAS, calculating the Hscore, and the current recommended treatment protocol.

**Disclosure: V. Kakkar:** None. **N. Dadiras:** None. **B. Szebenyi:** None. **T. Gillott:** None. **A. Alvi:** None.

